# Supplementary material for: Seed structure and phosphorylation in the fuzzy coat impact tau seeding competency
Source: Nat Commun. 2025 Oct 17;16:9240. doi: 10.1038/s41467-025-64312-2 (PMC12534512; doi:10.1038/s41467-025-64312-2)

Supplementary Materials for:

Seed structure and phosphorylation in the fuzzy coat impact tau seeding competency

Alysa Kasen[1], Sofia Lövestam[2], Libby Breton[1], Lindsay Meyerdirk[1], Jacob Alec McPhail[3,4], Kristin Piche[3], Ariel Louwrier[3], Colt D. Capan[1,5], Hyoungjoo Lee[1,5], Michel Goedert[2], Sjors H. W. Scheres[2], Michael X. Henderson[1]*

[1]Department of Neurodegenerative Science, Van Andel Institute, Grand Rapids, MI 49503
[2]MRC Laboratory of Molecular Biology, Cambridge, UK
[3]StressMarq Biosciences Inc., Victoria BC, Canada
[4]Institute for Neurodegenerative Disease, Weill Institute for Neurosciences, University of California, San Francisco, CA, USA
[5]Mass Spectrometry Core, Van Andel Institute, Grand Rapids, MI, 49503

*Correspondence:
Michael X. Henderson
michael.henderson@vai.org

Supplemental Table 1

Antibody	Vendor	Host	Catalog Number	Dilution	Use
Anti-Streptomyces griseus Pronase	LS Bio	Rabbit	LS-C147534	1:2,000	WB
Anti-Tau, Clone T49	Millipore Sigma	Mouse	MABN827	1:1,500	ICC
AT8	Thermo Fisher	Mouse		1:1,000<br>1:500	IHC<br>WB
Goat anti-Mouse IgG (H+L) Highly Cross-Adsorbed Secondary Antibody, Alexa Fluor™ Plus 680	Thermo Fisher	Goat	A32729	1:10,000	WB
Goat anti-Rabbit IgG (H+L) Highly Cross-Adsorbed Secondary Antibody, Alexa Fluor™ Plus 680	Thermo Fisher	Goat	A32734	1:10,000	WB
Goat anti-Rabbit IgG (H+L) Highly Cross-Adsorbed Secondary Antibody, Alexa Fluor™ Plus 800	Thermo Fisher	Goat	A32735	1:10,000	WB
Goat anti-Mouse IgG (H+L) Highly Cross-Adsorbed Secondary Antibody, Alexa Fluor™ Plus 800	Thermo Fisher	Goat	A32730	1:10,000	WB
Alexa Fluor 546 Goat Anti-Mouse IgG (H+L)	Thermo Fisher	Goat	A11003	1:500	ICC
Goat Anti-Rabbit IgG Biotinylated	Vector Laboratories	Goat	BA-1000-1.5	1:1,000	IHC
PHF1 (Supernatant)	Peter Davies	Mouse	AB_2315150	1:250	WB
Phospho-Tau Ser262	Thermo Fisher	Rabbit	44-750G	1:500	WB
Recombinant Anti-Mouse IgG1	Abcam	Rabbit	ab190481	1:1,000	IHC
Recombinant Anti-Tau EP2456Y	Abcam	Rabbit	ab76128	1:2,000	WB
Tau 640-680	Osenses	Rabbit	OST00329W	1:2,000	WB
Tau5	Binder/Kanaan	Mouse	AB_2721194	1:500	WB

Supplementary Table 1. Table of antibodies used. Abbreviations: Immunohistochemistry (IHC), Western Blot (WB), Immunocytochemistry (ICC)

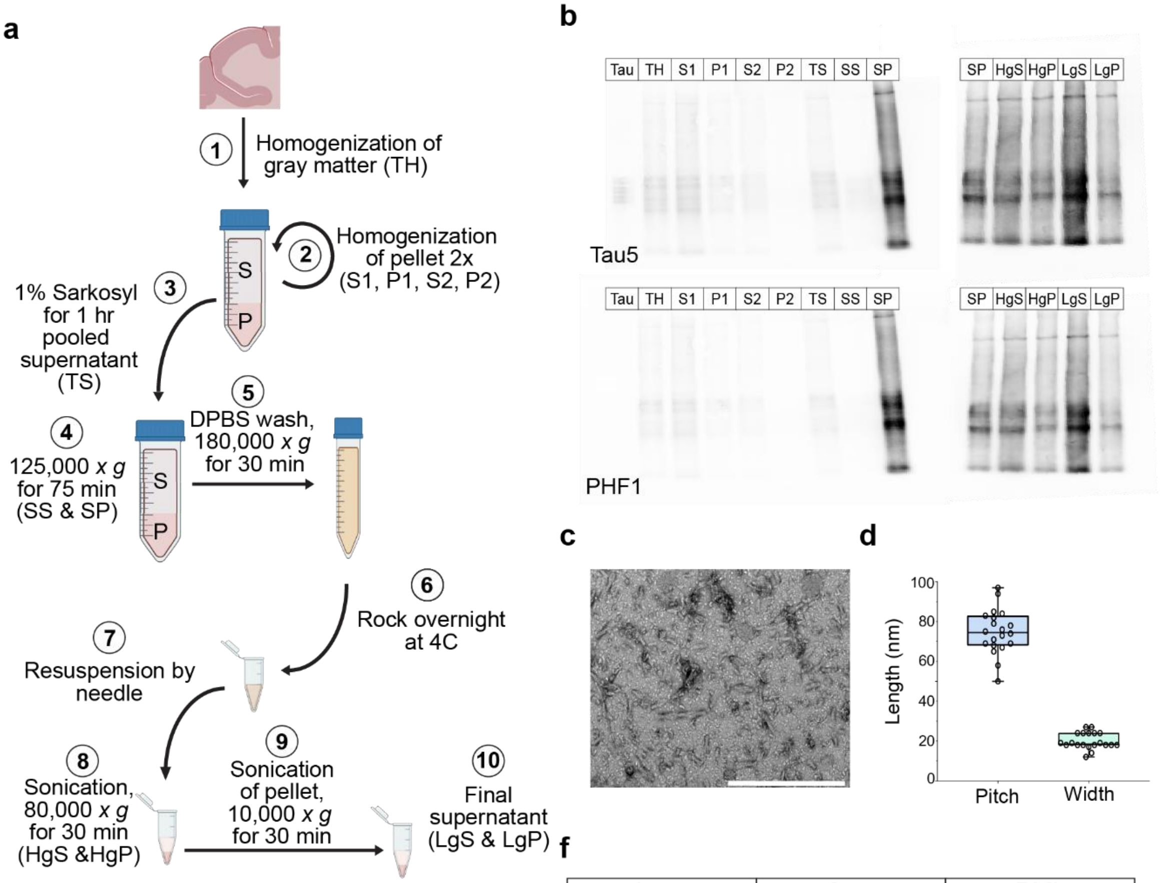

e

Total Protein	Tau Protein	Tau Percentage
13.4 mg/mL	2.8 mg/mL	20.9%

f

Age	Sex	PMI
77	F	15.1 hours
65	F	22 hours
75	F	17.14 hours

Supplementary Figure 1. Preparation and characterization of AD Tau. (a) Schematic of isolation of AD tau from cortical brain tissue. (b) Western blots of saved fractions from tau isolation for Tau5 (top) and PHF1 (bottom) compared to a standard tau ladder (Tau). TH – total homogenate, S1 – supernatant 1, P1- pellet 1, S2 – supernatant 2, P2 – pellet 2, TS – total supernatant, SS – sarkosyl supernatant, SP – sarkosyl pellet, HgS – high *g* spin supernatant, HgP – high *g* spin pellet, LgS – low *g* spin supernatant, LgP – low *g* spin pellet. (c) Representative TEM image of isolated AD tau used in this study. Scale bar 0.5 µm. (d) Measured width and pitch of AD tau fibrils from 20 fibrils across five images. Source data are provided as a Source Data file. (e) Table of protein concentration of the AD tau preparation. (f) Table of sample information for pooled brain samples used in the AD tau preparation. Created in BioRender. Kasen, A. (2025) https://BioRender.com/h8m20ur.

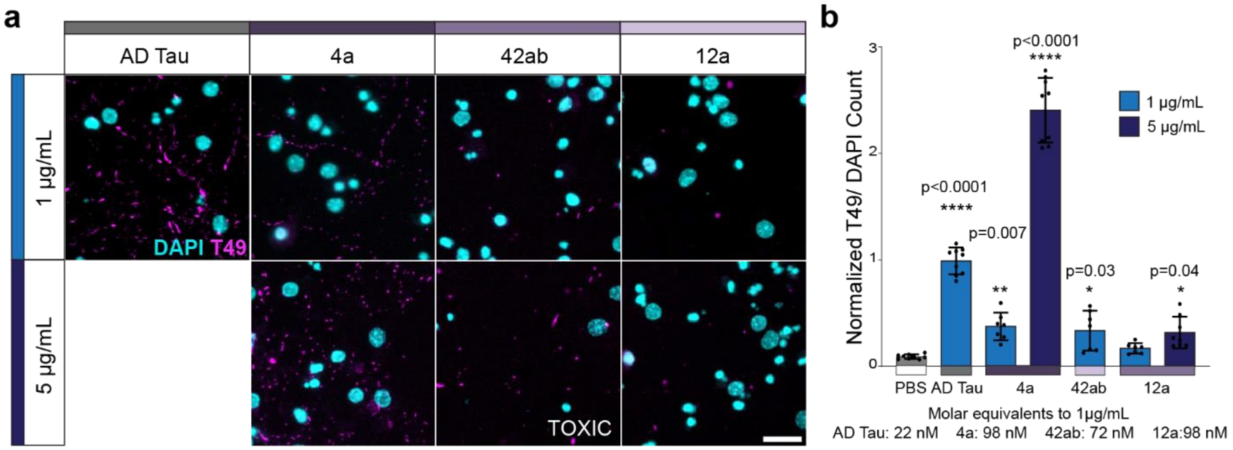

Supplementary Figure 2. Structurally-defined recombinant tau fibrils induce differential pathology in primary hippocampal neurons. (a) Representative images of primary hippocampal neurons treated with sonicated tau fibrils at 2 doses and maintained in culture for 21 days after fibril treatment. Scale = 20 µm. (b) Quantification of tau pathology, measured by T49, relative to DAPI count. Relative molar concentrations shown below graph. Data is presented as mean ± SEM with individual values plotted. N= 9 independent wells from 3 separate cultures, Welch ANOVA test and Dunnett's T3 multiple comparison test compared to PBS control. Source data are provided as a Source Data file.

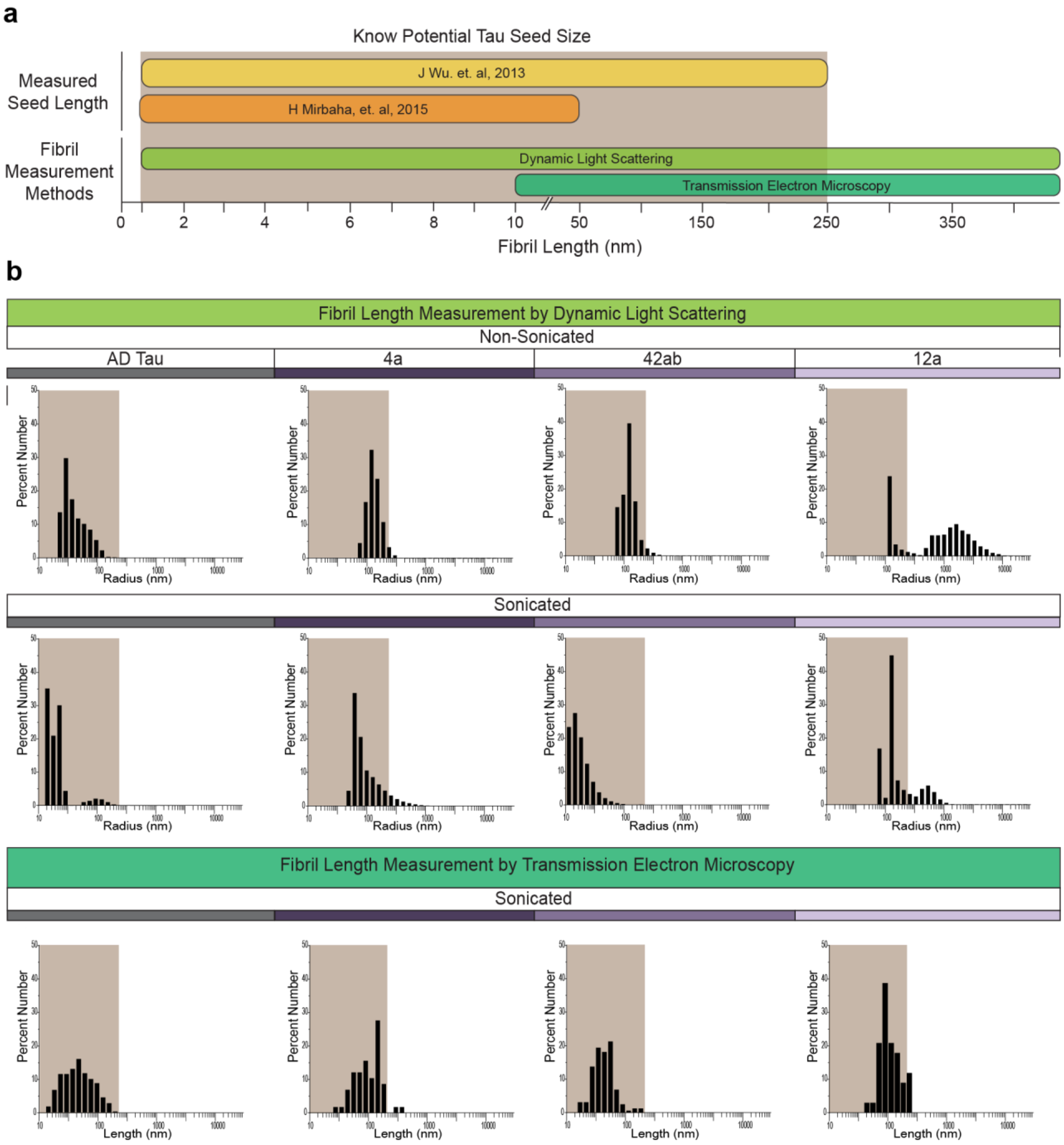

Supplementary Figure 3. Measurements of seed size in select fibrils. (a) Graph showing known effective tau seed size from published literature and the detection range of fibril size of dynamic light scattering (DLS) and transmission electron microscopy (TEM). (b) Plotted measured fibril size from DLS for non-sonicated and sonicated fibrils and from TEM for sonicated tau fibrils. Tan box highlights the known range of effective seed size. DLS data: n=3. TEM data: PHF n = 401, 4a, n=63, 42ab = 160, 12a n= 43. Fibrils measured across 30 images from each grid with a minimum size measurement of 15 nm.

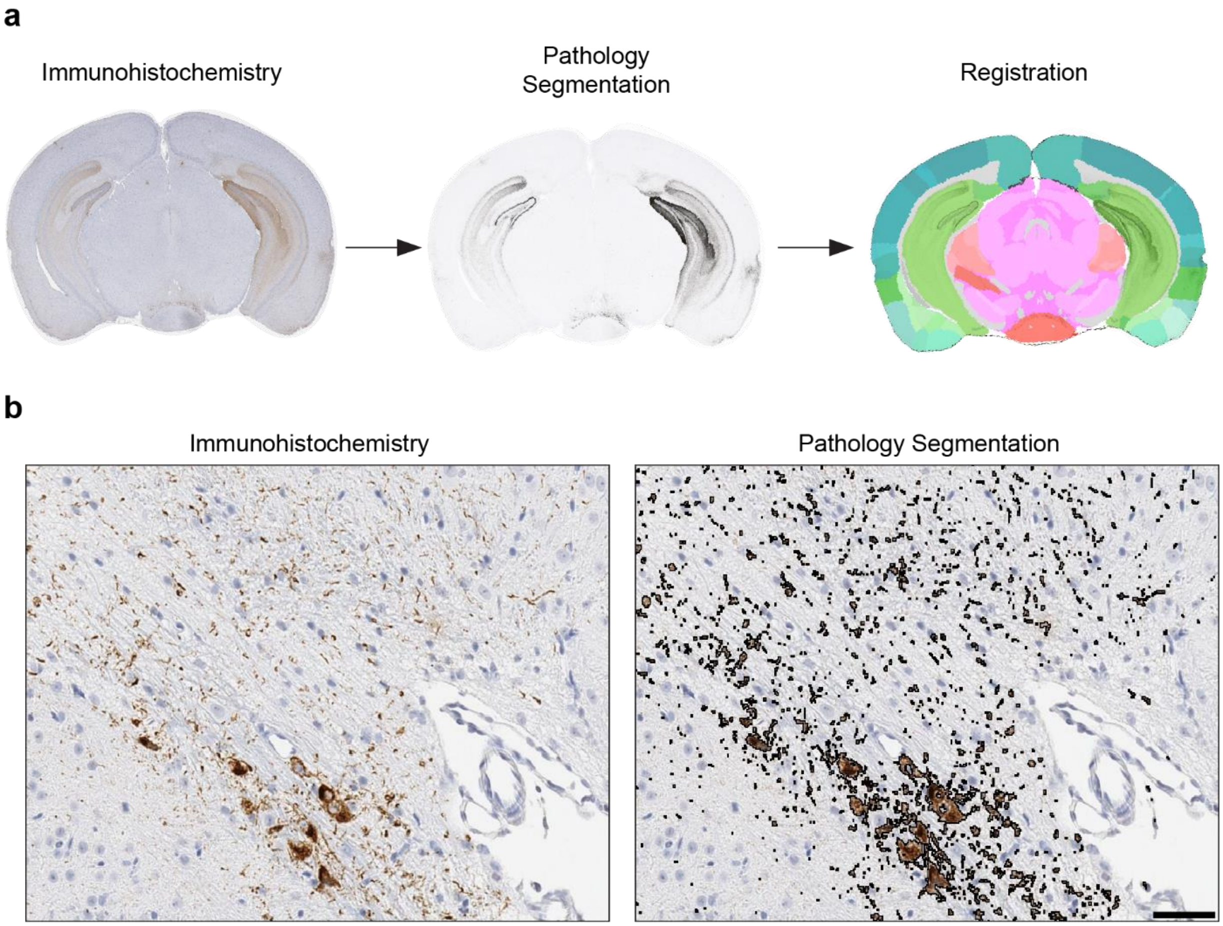

Supplementary Figure 4. Representative schematic of QUINT workflow. (a) Representative image of immunohistochemistry stain for AT8, labeled as immunohistochemistry, pixels positive for AT8 shown in black, labeled as pathology segmentation, and an overlay of the Allen Brain Atlas to the pathology segmentation, labeled as registration. (b) Representative positive pixel detection for AT8 in the hippocampus. Scale bar = 50 mm.

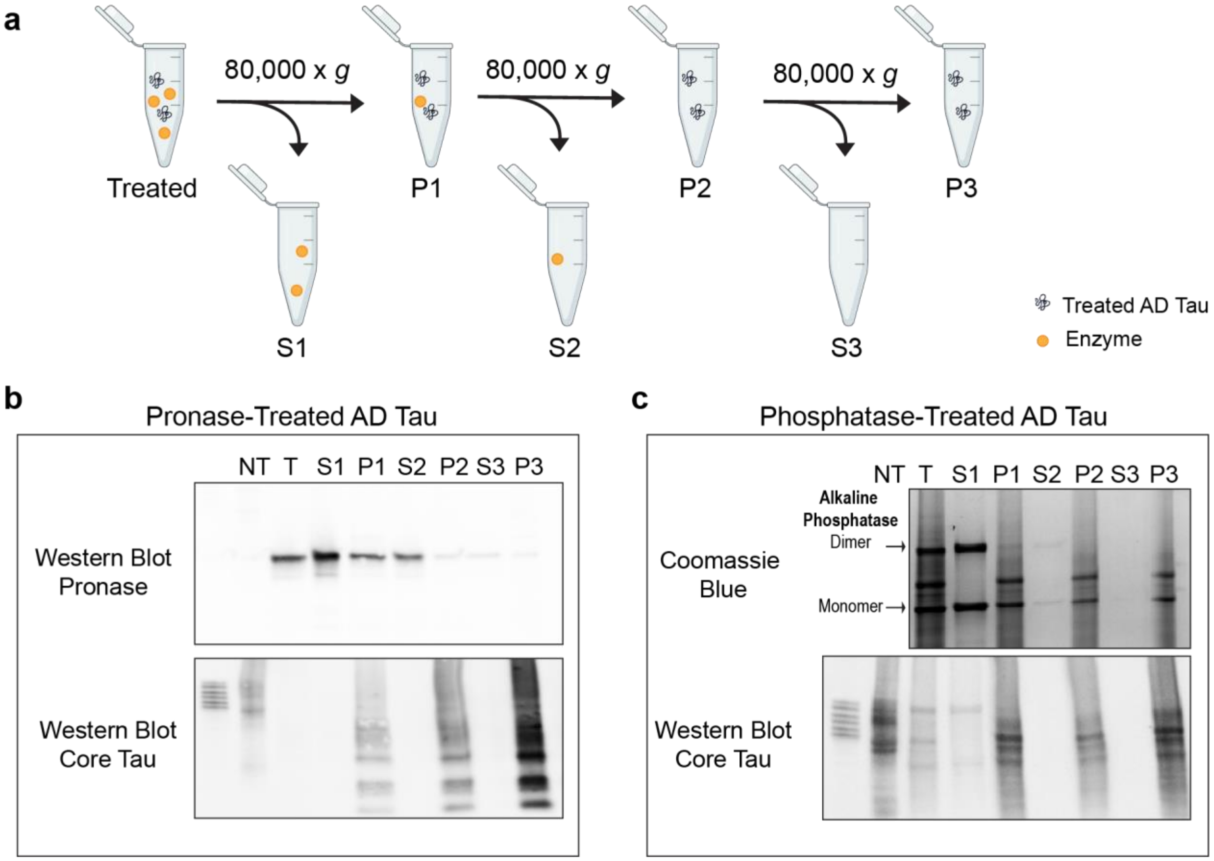

Supplementary Figure 5. Clean-up of modified tau fibrils. (a) Schematic of removal of enzymes used in the modification of AD tau. (b) Western blot of fractions from washes of pronase-treated AD tau for pronase (top) and OST00329W (bottom). (c) Coomassie blue gel (top) and Western blot for OST00329W (bottom) of fraction from phosphatase-treated AD tau. Created in BioRender. Kasen, A. (2025) https://BioRender.com/h8m20ur.

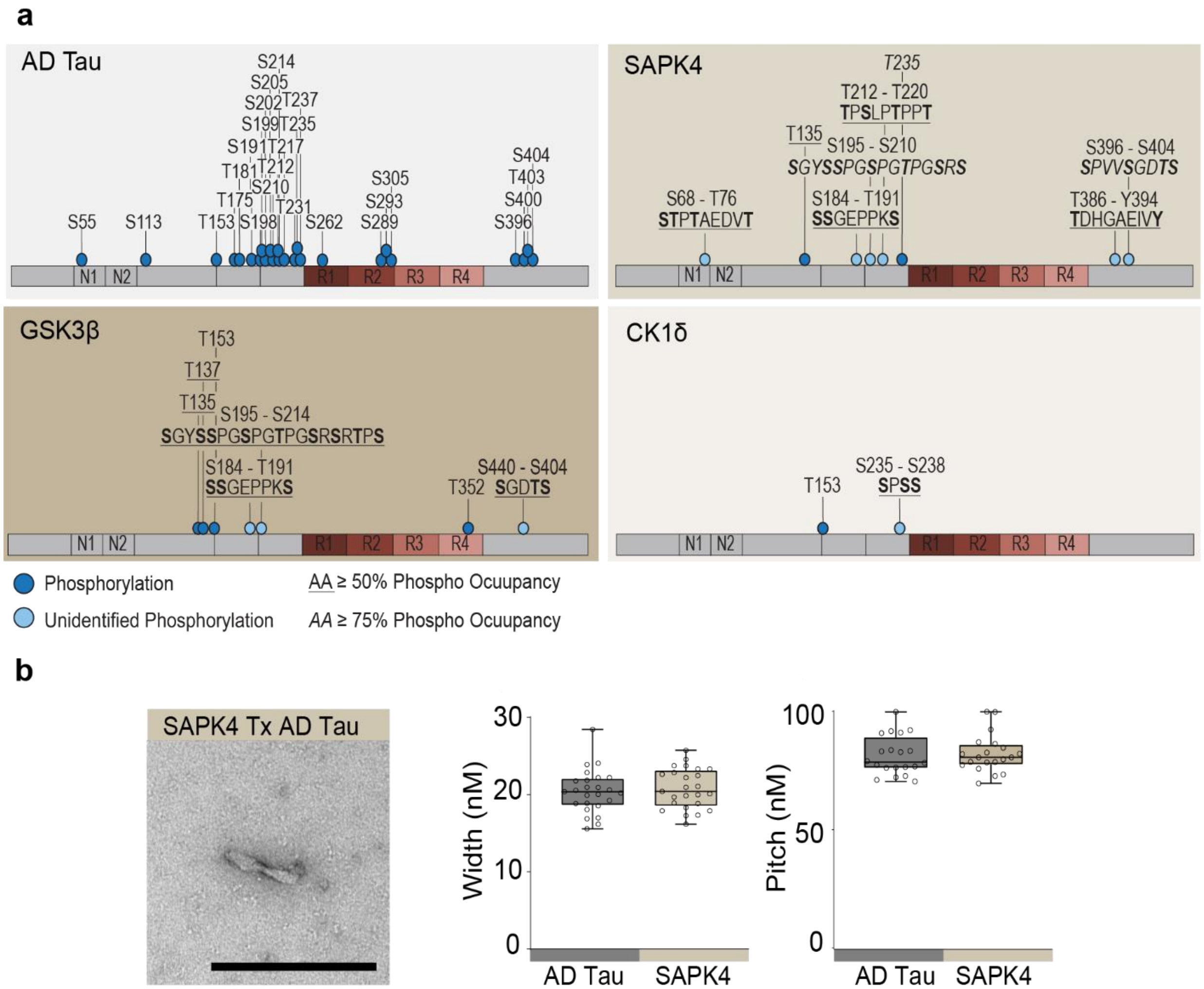

Supplementary Figure 6. Phospho-proteomic analysis following phosphorylation of tau monomer. (a) Schematic of phosphorylation sites reported on AD PHFs[9], and phosphorylation of tau monomer by SAPK4 (top right), GSK3β (bottom left), and CK1δ (bottom right). Single phosphorylation sites are marked by a dark blue dot and an unidentified phosphorylation site is indicated by a light blue. For each unidentified phosphorylation site, the sequence is noted with the possible phosphorylated amino acid in bold. Amino acids with greater than 50% phosphor-occupancy are underlined. Amino acids with greater than 75% phospho-occupancy are italicized. High-resolution LC-MS/MS was employed to unbiasedly identify phosphorylation sites. (b) Representative negative stain transmission electron microscopy (TEM) of SAPK4-treated AD tau. Quantification of measured width and pitch of AD tau and SAPK4-treated AD tau filaments measured in ImageJ. Individual filaments are represented by a single point width N= 25, pitch N= 20 . Source data are provided as a Source Data file.

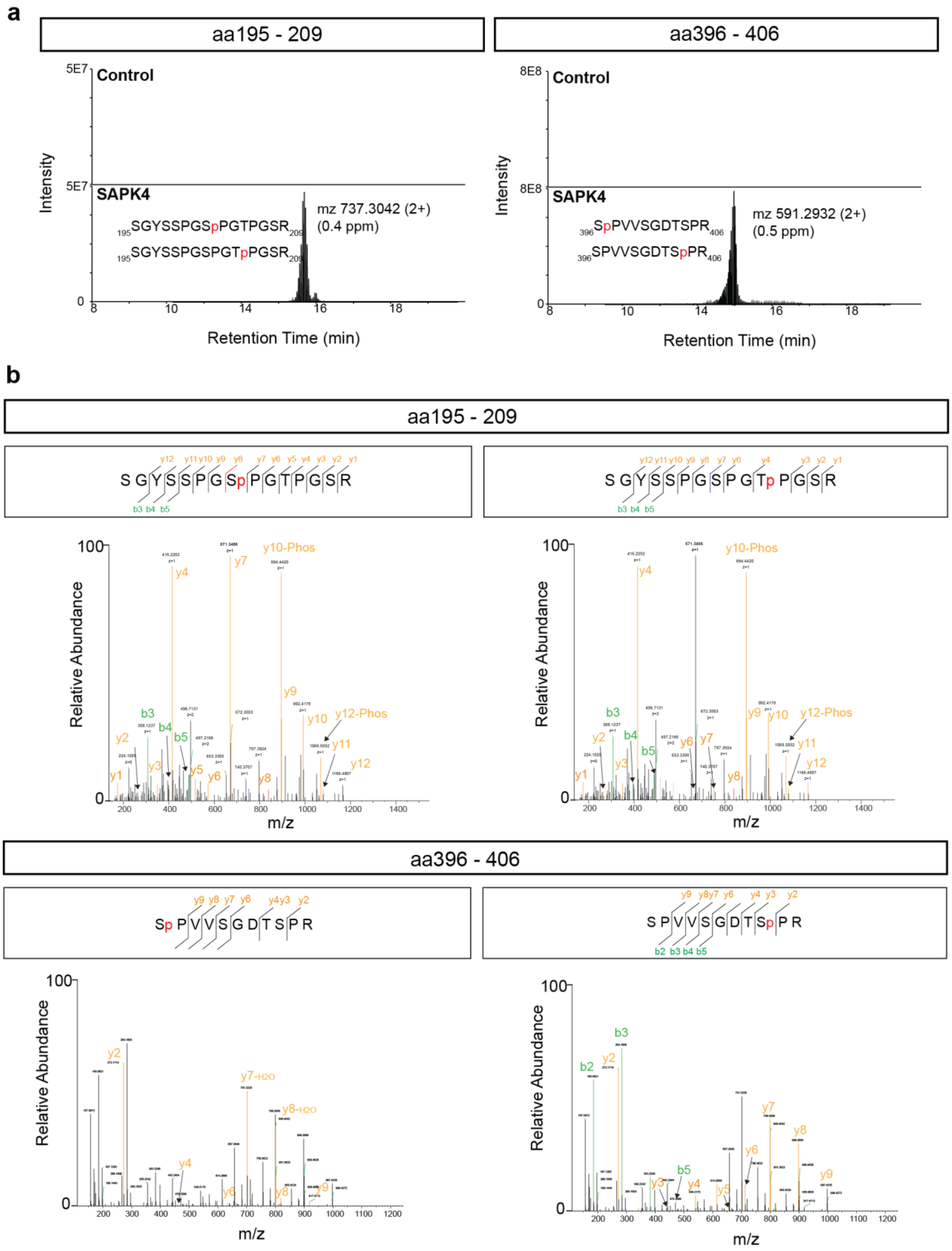

Supplementary Figure 7. Phospho-proteomic measurements of select peptides. (a) Relative abundance of the corresponding phosphopeptides in SAPK4 versus control samples. Isobaric peptides were co-

eluted and phosphopeptides were only detected in the SAPK4 samples, with precursor mass accuracy within 1 ppm. (b) MS2 spectra were manually interpreted and fragment ions were confidently assigned to support site localization.

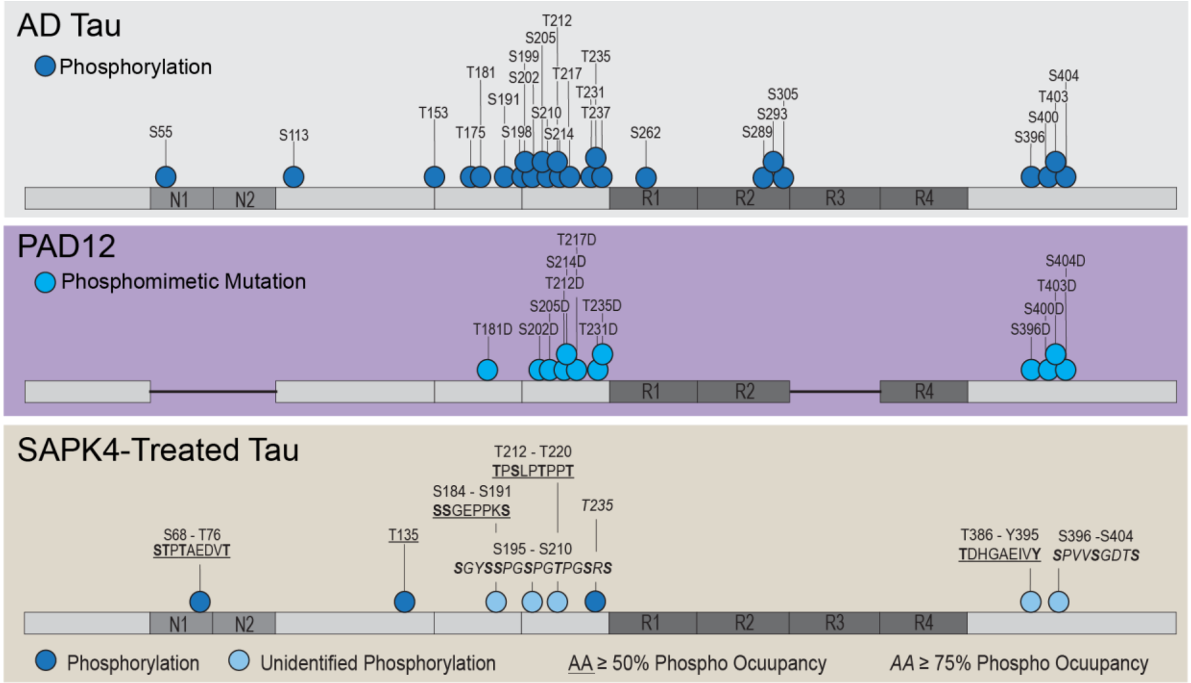

Supplementary Figure 8. Comparison of AD tau, PAD12 tau and SAPK4-treated tau. Schematics of phosphorylation sites reported on AD PHFs[9], phosphomimetic sites on PAD12, and phosphorylation of tau monomer by SAPK4. The phosphomimetic sites surrounding the AD tau core in PAD12 filaments are sufficient to show near full recapitulation of the seeding of AD tau, while re-phosphorylation with SAPK4 partially rescues seeding capacity. Taken together, this suggests that the while each cluster of phospho-sites contribute to increasing seeding capacity, phosphorylation both around the S202-T235 sites as well as S396-S404 are necessary to capture full seeding capacity of AD tau.

Supplementary Figure 1b Western Blots

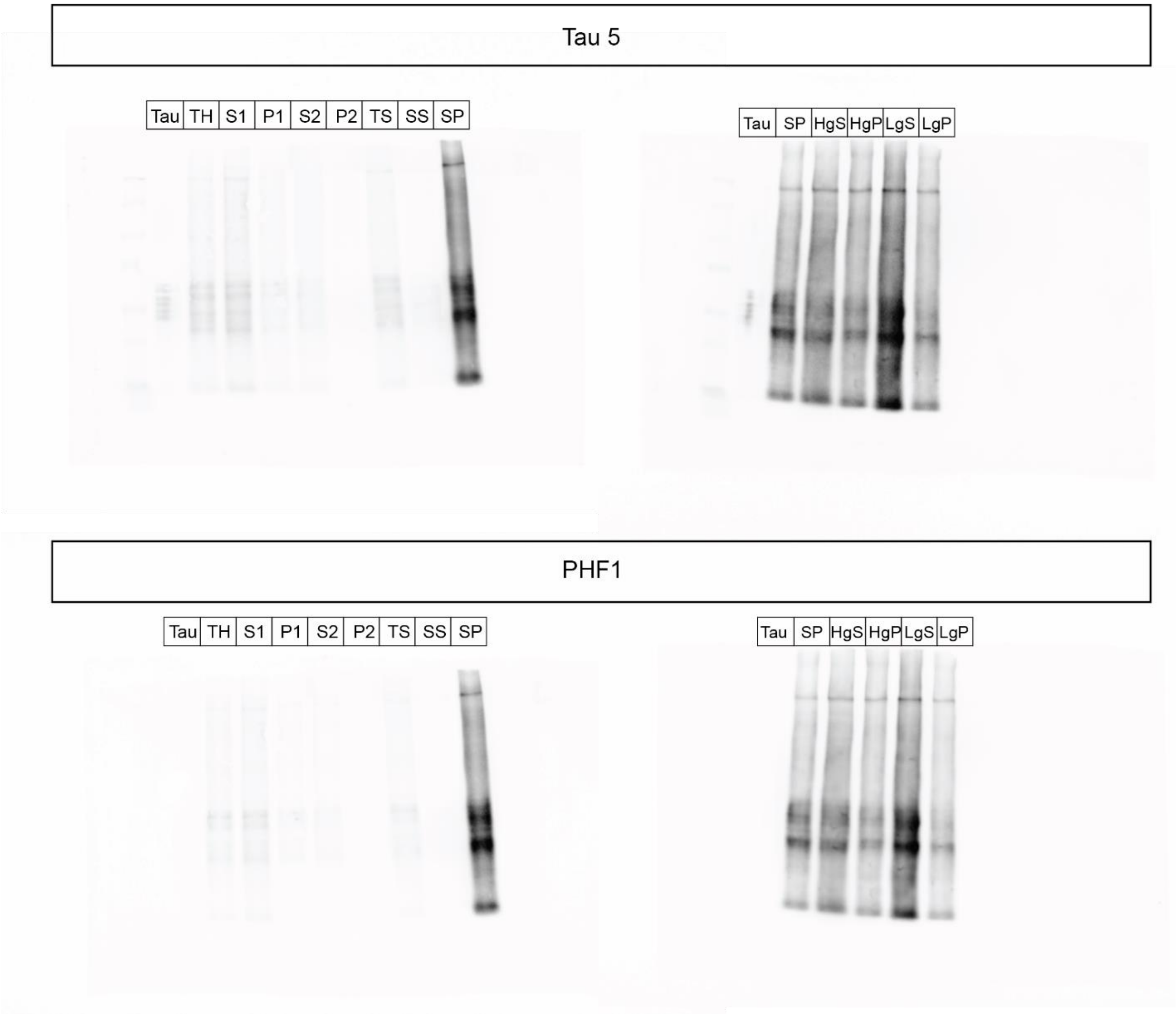

Supplementary Figure 5 Western blots and Gels

Supplementary Figure 5b

Western Blot: CoreTau

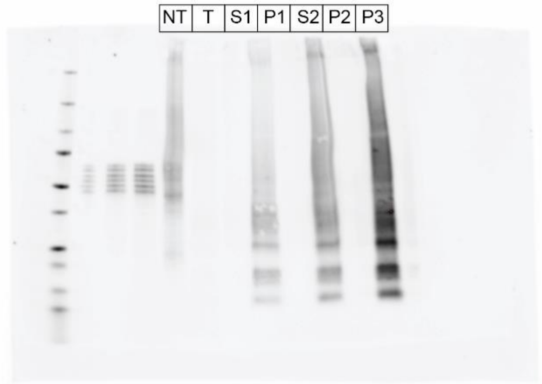

Western Blot: Pronase

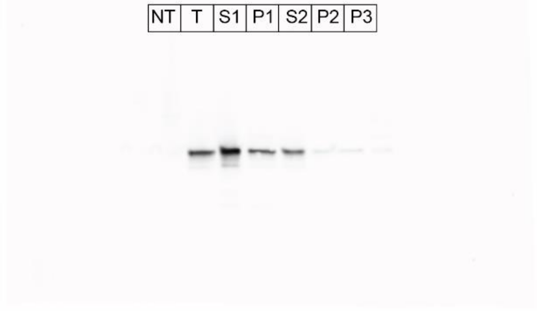

Supplementary Figure 5c

Protein Gel: Coomassie Blue

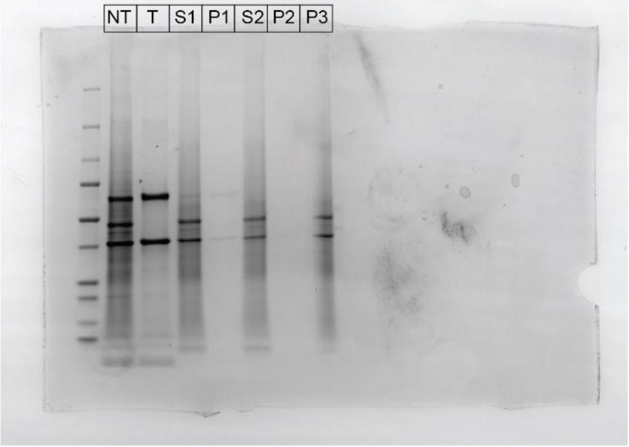

Western Blot: Core Tau

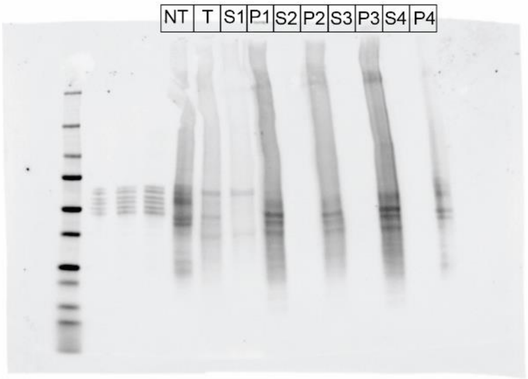

Supplement: Supplementary file 1 — Supplementary Information [file 41467_2025_64312_MOESM1_ESM.pdf]
