# Supplementary material for: Seed structure and phosphorylation in the fuzzy coat impact tau seeding competency
Source: Nat Commun. 2025 Oct 17;16:9240. doi: 10.1038/s41467-025-64312-2 (PMC12534512; doi:10.1038/s41467-025-64312-2)
Supplement: Supplementary file 2 — Reporting Summary [file 41467_2025_64312_MOESM2_ESM.pdf]

nature portfolio

Corresponding author(s): Michael X. Henderson

Last updated by author(s): Aug 7, 2025

Reporting Summary

Nature Portfolio wishes to improve the reproducibility of the work that we publish. This form provides structure for consistency and transparency in reporting. For further information on Nature Portfolio policies, see our Editorial Policies and the Editorial Policy Checklist.

Statistics

For all statistical analyses, confirm that the following items are present in the figure legend, table legend, main text, or Methods section.

n/a	Confirmed	
☐	☒	The exact sample size (n) for each experimental group/condition, given as a discrete number and unit of measurement
☐	☒	A statement on whether measurements were taken from distinct samples or whether the same sample was measured repeatedly
☐	☒	The statistical test(s) used AND whether they are one- or two-sided<br>*Only common tests should be described solely by name; describe more complex techniques in the Methods section.*
☒	☐	A description of all covariates tested
☐	☒	A description of any assumptions or corrections, such as tests of normality and adjustment for multiple comparisons
☐	☒	A full description of the statistical parameters including central tendency (e.g. means) or other basic estimates (e.g. regression coefficient) AND variation (e.g. standard deviation) or associated estimates of uncertainty (e.g. confidence intervals)
☐	☒	For null hypothesis testing, the test statistic (e.g. F, t, r) with confidence intervals, effect sizes, degrees of freedom and P value noted<br>*Give P values as exact values whenever suitable.*
☒	☐	For Bayesian analysis, information on the choice of priors and Markov chain Monte Carlo settings
☒	☐	For hierarchical and complex designs, identification of the appropriate level for tests and full reporting of outcomes
☒	☐	Estimates of effect sizes (e.g. Cohen's d, Pearson's r), indicating how they were calculated

Our web collection on statistics for biologists contains articles on many of the points above.

Software and code

Policy information about availability of computer code

Data collection	Dynamics 7.10.1.21<br>QuPath v.050 (RRID: SCR_018257)<br>QuickNII (RRID: SCR_016854)<br>VisuAlign (RRID: SCR_017978)<br>Nutil (RRID: SCR_017183)
Data analysis	Mouse Brain Heatmap (dx.doi.org/10.17504/protocols.io.n2bvj31jblk5/v1)

For manuscripts utilizing custom algorithms or software that are central to the research but not yet described in published literature, software must be made available to editors and reviewers. We strongly encourage code deposition in a community repository (e.g. GitHub). See the Nature Portfolio guidelines for submitting code & software for further information.

Data

Policy information about availability of data

All manuscripts must include a data availability statement. This statement should provide the following information, where applicable:

- Accession codes, unique identifiers, or web links for publicly available datasets
- A description of any restrictions on data availability
- For clinical datasets or third party data, please ensure that the statement adheres to our policy

Data presented in the manuscript is available on zenodo (https://doi.org/10.5281/zenodo.15090531). The mass spectrometry proteomics data have been deposited to the ProteomeXchange Consortium via the PRIDE (PubMed ID: 39494541) partner repository with the dataset identifier PXD062343. Reviewers can access the project with the following token: gEab65a9GxDp.

Research involving human participants, their data, or biological material

Policy information about studies with human participants or human data. See also policy information about sex, gender (identity/presentation), and sexual orientation and race, ethnicity and racism.

Reporting on sex and gender	*Use the terms sex (biological attribute) and gender (shaped by social and cultural circumstances) carefully in order to avoid confusing both terms. Indicate if findings apply to only one sex or gender; describe whether sex and gender were considered in study design; whether sex and/or gender was determined based on self-reporting or assigned and methods used.* *Provide in the source data disaggregated sex and gender data, where this information has been collected, and if consent has been obtained for sharing of individual-level data; provide overall numbers in this Reporting Summary. Please state if this information has not been collected.* *Report sex- and gender-based analyses where performed, justify reasons for lack of sex- and gender-based analysis.*
Reporting on race, ethnicity, or other socially relevant groupings	*Please specify the socially constructed or socially relevant categorization variable(s) used in your manuscript and explain why they were used. Please note that such variables should not be used as proxies for other socially constructed/relevant variables (for example, race or ethnicity should not be used as a proxy for socioeconomic status).* *Provide clear definitions of the relevant terms used, how they were provided (by the participants/respondents, the researchers, or third parties), and the method(s) used to classify people into the different categories (e.g. self-report, census or administrative data, social media data, etc.)* *Please provide details about how you controlled for confounding variables in your analyses.*
Population characteristics	*Describe the covariate-relevant population characteristics of the human research participants (e.g. age, genotypic information, past and current diagnosis and treatment categories). If you filled out the behavioural & social sciences study design questions and have nothing to add here, write "See above."*
Recruitment	*Describe how participants were recruited. Outline any potential self-selection bias or other biases that may be present and how these are likely to impact results.*
Ethics oversight	*Identify the organization(s) that approved the study protocol.*

Note that full information on the approval of the study protocol must also be provided in the manuscript.

Field-specific reporting

Please select the one below that is the best fit for your research. If you are not sure, read the appropriate sections before making your selection.

[x] Life sciences   [ ] Behavioural & social sciences   [ ] Ecological, evolutionary & environmental sciences

For a reference copy of the document with all sections, see nature.com/documents/nr-reporting-summary-flat.pdf

Life sciences study design

All studies must disclose on these points even when the disclosure is negative.

Sample size	For the in-vitro work, our pilot data allowed us to estimate three biological replicates would have greater than 80% power to detect a 1.2 fold difference between any two groups while maintaining a false discovery rate of less than 5%. For the in-vivo studies, previous work in the lab allowed us to estimate that using cohorts of 8 mice would give us greater than 80% power to detect a 1.5 fold difference between any two groups.
Data exclusions	For in-vitro work, samples were excluded that had greater that 25% cell death in the well. This criteria was set prior to analysis.
Replication	In-vitro work had both biological across a single experiment and the experiment was repeated at least 3 times. In vivo work was done across eight mice and analysis included two technical replicates for each brain region.

Randomization	For primary neuron experiments, treatments were designated multiple spots across a plate to minimize positional artifacts. For in vivo experiments, mice were assigned cohorts that were balanced for sex from a limited number of breeding schemes and littermates were balanced across treatment groups.
Blinding	Cell culture experiments were subjected to automated image acquisition and analysis with no user input and all images were included for final analysis. For in vivo work, images were blinded as they were brought into QuPath image analysis software using a blinding feature.

Reporting for specific materials, systems and methods

We require information from authors about some types of materials, experimental systems and methods used in many studies. Here, indicate whether each material, system or method listed is relevant to your study. If you are not sure if a list item applies to your research, read the appropriate section before selecting a response.

Materials & experimental systems

n/a	Involved in the study	
☐	☒	Antibodies
☒	☐	Eukaryotic cell lines
☒	☐	Palaeontology and archaeology
☐	☒	Animals and other organisms
☒	☐	Clinical data
☒	☐	Dual use research of concern
☒	☐	Plants

Methods

n/a	Involved in the study	
☒	☐	ChIP-seq
☒	☐	Flow cytometry
☒	☐	MRI-based neuroimaging

Antibodies

Antibodies used	AntiStreptomyces griseus Pronase; LS Bio; LS-C147534<br>Anti-Tau, Clone T49; Millipore Sigma; MABN827<br>AT8; Thermo Fisher; MN1020<br>Goat anti-Mouse IgG (H+L) Highly Cross-Adsorbed Secondary Antibody, Alexa Fluor™ Plus 680; Thermo Fisher; A32729<br>Goat anti-Rabbit IgG (H+L) Highly Cross-Adsorbed Secondary Antibody, Alexa Fluor™ Plus 680; Thermo Fisher; A32734<br>Goat anti-Rabbit IgG (H+L) Highly Cross-Adsorbed Secondary Antibody, Alexa Fluor™ Plus 800; Thermo Fisher; A32735<br>Goat anti-Mouse IgG (H+L) Highly Cross-Adsorbed Secondary Antibody, Alexa Fluor™ Plus 800; Thermo Fisher; A32730<br>Alexa Fluor 546 Goat Anti-Mouse IgG (H+L); Thermo Fisher; A11003<br>Goat Anti-Rabbit IgG Biotinylated; Vector Laboratories; BA-1000-1.5<br>PHF1 (Supernatant); Gift - Peter Davies<br>Phospho-Tau Ser262; Thermo Fisher; 44-750G<br>Recombinant Anti-Mouse IgG1; Abcam; ab190481<br>Recombinant Anti-Tau EP2456Y; Abcam; ab76128<br>Tau 640-680; Osenses; OST00329W<br>Tau5; Gift - Binder/Kanaan
Validation	All validations are available through the supplier.

Animals and other research organisms

Policy information about studies involving animals; ARRIVE guidelines recommended for reporting animal research, and Sex and Gender in Research

Laboratory animals	Mouse; C57BL/6J; injected at 3 months old<br>Mouse; CD-1; E18<br>Mouse; 6.Cg-Mapttm1.1(MAPT)Tcs; injected at 3 months old
Wild animals	Study did not involve wild animals
Reporting on sex	All mouse cohorts were balanced for sex. No sex differences were reported in this work.
Field-collected samples	Study did not involve samples collected from the field
Ethics oversight	All housing, breeding, and procedures were performed according to the NIH Guide for the Care and Use of Experimental Animals and approved by the Van Andel Institute Institutional Animal Care and Use Committee.

Note that full information on the approval of the study protocol must also be provided in the manuscript.

Plants

Seed stocks	*Report on the source of all seed stocks or other plant material used. If applicable, state the seed stock centre and catalogue number. If plant specimens were collected from the field, describe the collection location, date and sampling procedures.*
Novel plant genotypes	*Describe the methods by which all novel plant genotypes were produced. This includes those generated by transgenic approaches, gene editing, chemical/radiation-based mutagenesis and hybridization. For transgenic lines, describe the transformation method, the number of independent lines analyzed and the generation upon which experiments were performed. For gene-edited lines, describe the editor used, the endogenous sequence targeted for editing, the targeting guide RNA sequence (if applicable) and how the editor was applied.*
Authentication	*Describe any authentication procedures for each seed stock used or novel genotype generated. Describe any experiments used to assess the effect of a mutation and, where applicable, how potential secondary effects (e.g. second site T-DNA insertions, mosiacism, off-target gene editing) were examined.*
